# Child-Birth: Perineal Tears, Forceps, Chloroform, Massage

**Published:** 1914-02

**Authors:** E. C. Cartledge

**Affiliations:** Atlanta, Ga.


### CHILD-BIRTH: PERINEAL TEARS, FORCEPS, CHLOROFORM, MASSAGE.

By E. C. Cartledge, M. D., Atlanta, Ga.

My chief thought and purpose in this paper is the prevention of torn perinei at labor. I think there is one best forceps only, so far is I've seen, and that is Dewee's which I pass among you for inspection by any not familiar with it. It is the only true axis-traction forceps, not merely having the axis-traction handle, but also two opposed pivotal points indicating exactly when your forceps pull is in proper axis or not, at each point of the 90 degrees of pelvic strait curve. There is no heel to this forceps to increase perineal strain and tear: in fact, it should prevent tears by allowing control of head and proper retardation for perineal stretching. I have never seen the neccessity of removing the forceps until the head is entirely born. The thinness and fit of blades do not increase the head diameter, unless it be a very small head possibly. Perhaps the chief reason for not using the forceps in nearly every confinement case would be the objection to the more prolonged anaesthesia.

Now as to the use of Chloroform:

I will state parenthetically that I use Ether in repairing torn perinei and whenever that anaesthetic can be used.

From talking with others, from discussions and readings, it has appeared to me that I get a service from Chloroform not commonly appreciated and that there is a variableness in its use among different obstetricians. There are those who tell me they can use Chloroform to relieve the travail of the mother without interfering with labor's progress, with me, to arrest pain has always been to arrest labor. And so I resort to Chloroform only to save the perineum; and to do this exactly right and to accomplish the fullest possibilities, demands an exactness of the utmost nicety; and which, in my experience, can only be obtained by the Doctor himself administering the chloroform with his own free left hand, a watchful assistant holding the inhaler in place over the face, while the Doctor's gloved right hand, placed upon the advancing head and bulging perineum, judges, from the position and rapidity of advancement, just how few or how many drops of chloroform are needed, and if the patient received them upon the inhale or exhale of respiration, their under or over effect, how many drops and how fast to increase or decrease their supply, did the head so recede that we may have to omit chloroform one, two or more pains, or must we push the chloroform to measure to the non-receding on-coming advancement. It is being on the "qui vine" indeed to keep adjusted this constantly shifting balance. No two cases are just alike, it seems. Some need really no chloroform: Some prolonged chloroform of variable degrees of narcosis to procure a sufficiently slow dilatation to insure against a tear. I said "slow dilation." "Slow dilation" is the "golden text" of this lesson and of all this paper.

This time element to save tears in sphincter muscle and tissue stretching was first impressed upon me by my first case of anal dilatation for fissure ani, then the style of practice for such. The result was a local tragedy of mutilation and multiplied tears, and larger fissures made in order to cure the one small fissure. Subsequently, by going slowly enough, I have stretched and paralyzed the sphincter ani till my entire hand passed up between the tuberasities of ischium without a tear of skin or mucoso or gross muscle. The difference in the anus without a tear and the one stellate and twinkling in radiate tears was just the one element of time.

And so I believe we can reduce tears in our young mothers, or old, to a rarety. It is worth the Doctor's time and care, though he may be able to make a mechanically perfect restoration of the torn parts. The scar more easily tears subsequently as you know.

My last four cases taken inversely as to time or sequence illustrate some points:

First was seven months child, 4½ pounds, primipora; just as head began to put perineum on the slight stretch, pains quieted and advancement ceased for quite a few minutes. The nurse began giving the patient's lower spine a firm deep massage when suddenly a hard effective pain sent the head surging over the half dilated perineum producing a small tear, though the head was so small. Time did it.

Second case should never have been torn, it would seem: multipara, weight about 160, height about 5′ 10″. Baby, 9½ pounds, born before I arrived, tear nearly to rectum. Pains were few and effective. Time tore her, or rather lack of time.

Third and fourth cases were primiparae. Each baby 9½ pounds as was case two. Neither mother torn one particle, not a little slit in skin. They were 16 and 22 years old. Usually I do not expose the mother in delivery. I use my fingers, obstetrical eye, as my former and revered teacher, Dr. Virgil O. Harden used to express it; but this perineum of one aged 22 seemed so impossible of saving that I watched it with my eye and saw the perineum spread itself up over the bulging head into a brood thin as paper covering. Fortunately the chloroform held it just so far several minutes, without any move, against that straining barrier which each moment I expected to see split right down and back, until I dared hold the head there no longer, and I could hardly believe it when I saw the head, after even further stretching of perineum, come on over without a smallest nick in the skin or mucosa. Time did it! I know of no other legitimate means of retarding labor and saving the perineum than chloroform and forceps. These can be dosed and measured, so to speak, to the finest necessities of each case if you are alertly right on your guard with every poise of faculty. A tear may occupy only one moment or second of time, but that moment must be seen, anticipated and met.

As to the massage part of my subject:

I have little or no experience or information to offer you. The suggestion came to me when the pains so quickly and vigorously responded apparently to the spinal massage in the first case referred to. I looked up the item of uterine spinal reflexes in spandylotherapy of Abrams and reflexology of Dr. Arnold Snow, New York City, and find that they say that the stimulation of the 1st, 2nd and 3rd lumbar nerve roots will produce urine stimulation and contraction with dilation of cervix.

I take the idea for future experimentation or trial by myself and offer it to you.

Grant Building, Atlanta, Ga.

